# Psychological and Electrophysiological Correlates of Word Learning Success

**DOI:** 10.11621/pir2021.0111

**Published:** 2021-06-30

**Authors:** Nadezhda A. Mkrtychian, Svetlana N. Kostromina, Daria S. Gnedykh, Diana M. Tsvetova, Evgeny D. Blagovechtchenski, Yury Y. Shtyrov

**Affiliations:** a Laboratory of Behavioural Neurodynamics, St. Petersburg State University, St. Petersburg, Russia; b Center of Functionally Integrative Neuroscience (CFIN), Aarhus University, Department of Clinical Medicine, Aarhus, Denmark

**Keywords:** word learning, semantics, EEG, alpha oscillations, ERP, ambiguity tolerance, BIS/BAS scales

## Abstract

**Background:**

A rich vocabulary supports human achievements in socio-economic activities, education, and communication. It is therefore important to clarify the nature of language acquisition as a complex multidimensional process. However, both the psychological and neurophysiological mechanisms underpinning language learning, as well as the links between them, are still poorly understood.

**Objective:**

This study aims to explore the psychological and neurophysiological correlates of successful word acquisition in a person’s native language.

**Design:**

Thirty adults read sentences with novel nouns, following which the participants’ electroencephalograms were recorded during a word-reading task. Event- related potentials in response to novel words and alpha oscillation parameters (amplitude, variability, and long-range temporal correlation dynamics) were analyzed. Learning outcomes were assessed at the lexical and semantic levels. Psychological variables measured using Amthauer’s test (verbal abilities), BIS/BAS scales (motivation), and the MSTAT-1 (ambiguity tolerance) and alpha oscillation parameters were factored.

**Results:**

Better recognition of novel words was related to two factors which had high factor loadings for all measured alpha oscillation parameters, indicating the role of attention networks and respective neural activity for enabling information processing. More successful learners had lower P200 amplitude, which also suggests higher attention-system involvement. Another factor predicted better acquisition of word meanings for less ambiguity-tolerant students, while the factor which pooled logical conceptual thinking ability and persistence in goal-reaching, positively correlated with acquisition of both word forms and meanings.

**Conclusion:**

The psychological factors predominantly correlated with word-learning success in semantic tasks, while neurophysiological variables were linked to performance in the recognition task.

## Introduction

During their lifespans, human beings learn on average more than 40,000 words (Kuipers, Uminski, Green, Hughes, & Aglietti, 2017). Generally, word learning may be posited as including acquisition of novel word forms (phonological and/or orthographic), new meanings (both novel semantics and connections with previous semantic knowledge), and establishing links between them (Mkrtychian et al., 2019). The balance between these constituent parts depends on exact circumstances: for instance, learning a new meaning for familiar polysemic words does not include word form acquisition, whereas synonym or foreign word learning may only require connecting novel word forms to already familiar concepts, which have been previously established for the native language (L1).

While word-form learning has been investigated often, word-meaning acquisition remains largely understudied. In experimental settings, the connections between novel word forms and their meanings could be ascertained using pictures (Apfel-baum & McMurray, 2017; Bermúdez-Margaretto, Beltrán, Cuetos, & Domínguez, 2019); definitions (Bakker, Takashima, van Hell, Janzen, & McQueen, 2015; Liu & van Hell, 2020); or meaningful sentence contexts (Elgort, Brysbaert, Stevens, & Van Assche, 2018; Lauro, Schwartz, & Francis, 2020; Mestres-Missé, Rodriguez-Fornells, & Münte, 2007). In natural situations, new meanings are acquired either through expli cit instruction or by inferring them from their contexts (Jenkins & Dixon, 1983); the latter (contextual acquisition) is prevalent for L1 learning (Nagy, Herman, & Anderson, 1985). There are at least two interconnected cognitive mechanisms of contextual learning: associative learning and hypothesis testing (Yu & Smith, 2012). These mechanisms allow the retrieval of correct word-referent pairings from ambiguous learning contexts.

Successful word learning eventually provides one with a rich vocabulary, which supports one’s communication abilities and is key for achieving success in various social, educational, and professional fields. Any deficits which impede language learning negatively influence cognitive development and academic achievement. Therefore, it is important to investigate and understand the key factors determining success in novel word acquisition.

### External Factors

The external factors of word learning success include the learning materials and methods used. The existing literature on learning conditions has generally focused on second language (L2) learning, probably because L1 is usually acquired in a natural, implicit way, whereas L2 usually needs an explicit learning strategy (in monolingual environments). Arguably, the same factors could be important in terms of L1 learning, for instance, when studying new professional or scientific terminology. Thus, the method of learning (implicit or explicit) could be one of the factors determining learning success (Dickinson et al., 2019; Sobczak & Gaskell, 2019).

Learning outcomes are also affected by the modality of stimulus presentation (Penney, 1989). The results, however, are task-dependent and related to the appropriateness of the specific modality to the task (Welch, DutionHurt, & Warren, 1986). For instance, it has been demonstrated that spatial tasks involve the visual modality (*e.g*., Bertelson & Radeau, 1981; Jack & Thurlow, 1973; Kitagawa & Ichihara, 2002), whereas auditory tasks rely on temporal judgment (*e.g*., Fendrich & Corballis, 2001; Hansen & Cottrell, 2013; Welch et al., 1986).

Moreover, word learning outcomes are affected by the learners’ activity — vocal (oral), subvocal (silent), or written repetition (Candry, Deconinck, & Eyckmans, 2018; Junttila & Ylinen, 2020). However, this effect is ambiguous and depends on other factors (Zamuner, Strahm, Morin-Lessard, & Page, 2018). Thus, the advantage of vocal over silent rehearsal was found only for phonologically unfamiliar (but not native-language similar) words (Kaushanskaya & Yoo, 2011).

Finally, many other psycholinguistic variables, such as word length and frequency, number of lexical neighbors, concreteness, emotional validity, and imageability may affect word learning (Ferré, Ventura, Comesaña, & Fraga, 2015). Thus, experimental procedures involve a variety of external factors that have a great impact on word learning success. This makes it difficult to compare the results of different studies and, consequently, highlights the necessity of simultaneous employment of both psychological and neurophysiological approaches in a single study.

### Internal Factors

There is ample evidence of the influence of students’ psychological features on their learning achievement; these include the level of concentration, attention allocation and maintenance, short-term and motor memory, thinking skills, cognitive control, etc. (Chen & Chen, 2015; Kostromina, Mkrtychian, Kurmakaeva, & Gnedykh, 2017; York, Gibson, & Rankin, 2015; Zohar & Dori, 2003). As for language learning in particular, it has been shown that cognitive flexibility (Ehrman & Oxford, 1995), attention allocation (Schmidt, 2012), and working memory capacity (Kroll, Michael, Tokowicz, & Dufour, 2002) allow students to be more effective in L2 learning.

Crucially, most psychological studies dedicated to novel word acquisition relate to L2 learning. Such studies have, for instance, indicated the role of motivation (Gardner, Lalonde, & Moorcroft , 1985; Gömleksiz, 2001; Karlak & Velki, 2015; Sheu, 2016); anxiety (see Teimouri, Goetze, & Plonsky, 2019, for meta-analysis); tolerance of ambiguity (Genç, 2016); and risk-taking (Yulan & Yuewu, 2020). Motivation has been called “one of the important aspects of second language acquisition” (Anjomshoa & Sadighi, 2015, p. 135). Ambiguity tolerance also appears to facilitate L2 learning (Ghanizadeh & Allahdadi, 2015) and has been associated with various language learning achievements, as demonstrated in grammar, dictation, and speaking test results (Chapelle & Roberts, 1986). The least ambiguity-tolerant learners have also been shown to use more language learning strategies (Sadeghi & Soleimani, 2016). On the other hand, a recent study showed that tolerance of ambiguity did not correlate with vocabulary knowledge but rather had a relationship with self-perceived achievement in L2 vocabulary learning (Basöz, 2015).

As for L1 acquisition studies, they mostly concern native vocabulary learning in childhood. These studies have revealed relationships between vocabulary acquisition and working memory (Verhagen & Leseman, 2016), attention, (Bastianello, Majorano, & Burro, 2018) and other executive functions (White, Alexander, & Greenfield, 2017), as well as inference making (Kim, 2017) and thinking (Kostromina & Nagaeva, 2008). However, there is a lack of evidence of the influence of adult learners’ individual differences on word acquisition in L1.

### Neuropsychological Correlates

Along with the investigation of the psychological factors involved in language learning, there is a body of neurophysiological studies exploring the brain activity underpinning this process. Many of these were conducted using electroencephalography (EEG), one of the most popular and affordable methods for non-invasive assessment of brain activity. Due to its superb temporal resolution, an EEG is particularly well suited for studying the highly dynamic neural processes subserving the language function. Different electrophysiological measures which can be acquired using EEG are associated with specific psychological functions, and include event-related potentials (ERPs) (Kappenman & Luck, 2012) and oscillatory activity (Klimesch, 2012).

Since novel word learning assumes the acquisition of both new word forms and previously unfamiliar meanings, the ERP components of orthographic and semantic processing are of particular interest. The P200 (or P2) component with a frontocentral positive-going distribution is related to orthographic form recognition (Bermúdez-Margaretto, Beltrán, Shtyrov, Dominguez, & Cuetos, 2020), and its amplitude is correlated with word frequency (Y. Wang, Jiang, Huang, & Qiu, 2021). Moreover, a positive ERP around 200 ms is connected with a top-down control over attention (Morrison & Taler, 2020), and an increase in its amplitude is associated with a decrease in the level of attention (Cnudde et al., 2021).

Another important EEG marker for word processing is N400; this is the negative ERP traditionally associated with semantic processing (Kutas & Federmeier, 2011), which amplitude reduction indicates the integration of novel words into the lexico-semantic cognitive system. Interestingly, it has been shown that a rapidly developed N400 has frontal distribution, whereas, after a consolidation, it typically shifts into centro-parietal areas (Rasamimanana, Barbaroux, Colé, & Besson, 2020).

By measuring the oscillatory activity which is believed to reflect the functioning of large-scale neural networks, properties such as its frequency, amplitude, and the temporal dynamics of oscillatory patterns in specific frequency bands can be assessed. Alpha-band activity (oscillations in the 8-12 Hz range) is of particular interest since these oscillations reflect the general activation of the cerebral cortex and have been associated with many cognitive processes. The most important one is attention (Bastiaansen, Böcker, & Brunia, 2002; Klimesch, 1999), which, in turn, influences visual perception (Hanslmayr, Gross, Klimesch, & Shapiro, 2011), and consequently may affect the success of written word acquisition. Alpha-band oscillations reflect suppression of brain structures irrelevant to a specific task (Jensen & Mazaheri, 2010) and the selection of relevant information (Klimesch, 2012).

On e particularly interesting measure of oscillatory dynamics is the so-called long-range temporal correlation (LRTC). This approach to quantifying oscillatory patterns uses a type of autocorrelation analysis and has been successfully used to investigate a variety of important neural processes such as the excitation/inhibition ratio, system memory, and the efficiency of information transfer (Linkenkaer-Hansen, Nikouline, Palva, & Ilmoniemi, 2001; Linkenkaer-Hansen, Nikulin, Palva, Kaila, & Ilmoniemi, 2004; Linkenkaer-Hansen et al., 2007; Montez et al., 2009; Nikulin & Brismar, 2005; Palva et al., 2013; Smit, Linkenkaer-Hansen, & de Geus, 2013).

### Objective

As reviewed above, there are multiple factors at play which potentially affect success in word acquisition. However, there is still a lack of research investigating the psychological and neurophysiological correlates of language learning in an integrative fashion, which could help elucidate language acquisition as a complex multidimensional process.

Our study aimed to investigate both the electrophysiological and psychological correlates of contextual learning of new nouns in the native language. We hypothesized that more and less successful word learners differ in their verbal intelligence, motivation, and tolerance of ambiguity, as well as in their brain responses to novel words in an attention-demanding task.

To explore word learning success thoroughly, we chose five tasks to assess different levels of lexical and semantic word processing. The stimulus preparation, and learning and testing procedures, were developed according to recommendations for neurophysiological studies of language learning and word acquisition (Blagovechtchenski et al., 2019). We used EEG, a non-invasive neuroimaging method with a high temporal resolution, which makes it most appropriate for studying dynamic cognitive processes (Erickson, Kappenman, & Luck, 2018). We assessed the parameters of alpha oscillations, as they provide an objective indicator of the state of attention networks (Klimesch, 2012). The main characteristics of these oscillations (amplitude, variability, and long-range temporal correlations) were analyzed and linked to novel word learning performance.

Since the level of alpha-range activity reflects the level of attention and visual alertness (Bastiaansen et al., 2002; Stothart & Kazanina, 2013; J. Wang, Conder, Blitzer, & Shinkareva, 2010), we anticipated that the amplitude of alpha oscillation would be negatively correlated with word learning success.

It has been shown that fluctuations of ongoing brain oscillations are linked to variability in behavioral responses, particularly, in visual stimulus detection (Zazio, Schreiber, Miniussi, & Bortoletto, 2020). A decrease of alpha power implies a more liberal detection criterion, whereas the opposite is true for its increase (Iemi & Busch, 2018; Iemi, Chaumon, Crouzet, & Busch, 2017). Thus, high variability of alpha oscillation may be linked to more variable responses to the same stimuli. This, in turn, suggests a negative correlation of such variability and behavioral accuracy in time-limited attention-demanding tasks such as Recognition and Lexical decision.

For ERPs to newly learnt words, we expect to see differences in P200 and N400 amplitudes between more and less successful word learners since, for the latter, new nouns may remain orthographically (P200) and semantically (N400) less familiar.

## Methods

### Participants

Thirty right-handed healthy volunteers (M_age_ = 23.4 year; range = 18–35 years; 53.33% females), all monolingual Russian speakers, participated in the study. All subjects gave their written informed consent and filled out a questionnaire about their demographic characteristics and health. The study protocol was approved by the Ethical Committee of Saint Petersburg University.

### Stimuli

Novel words were simultaneously provided with both new word forms and novel meanings. To create novel word forms, four groups of 10 Russian nouns with the same structure (CVCCVCVC, where C is a consonant and V is a vowel) were chosen. The groups did not differ statistically in their lemma and last-syllable frequency. Novel word forms were created by mixing ultimate syllables within the group: for example, вурдалак ([vurdal’ak], eng. vampire) –> вурдак’ет* ([vurdak’et], a pseudoword). Thus, 40 novel word forms were produced. They were rotated across subjects in terms of their experimental role and were used as either novel words (concrete or abstract) or untrained fillers; moreover, novel word forms were assigned to the meanings in a counterbalanced fashion. Rare or obsolete objects (concrete semantics, 10 items) or abstract concepts borrowed from foreign cultures (10 items) were used for the novel meanings.

### Learning Procedure

**Figure 1.**
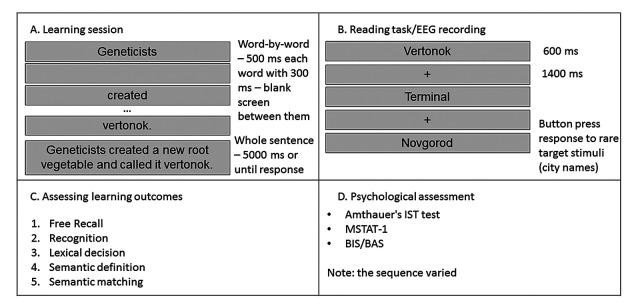
Experimental procedures: contextual learning session (A) was followed by EEG recording during a word reading task (B), behavioral assessment of learning success (C), and psychological assessment of the volunteers (D).

Each novel word was presented visually in five eight-word sentences (*Figure 1*), which gradually revealed its meaning from the described situational context. Every sentence was first presented word-by-word and then, to ensure understanding, completely on the computer screen. Participants had to read these sentences sitting in an acoustically and electrically shielded chamber and press a button after reading the whole sentence. Presentation of sentences was managed using NBS Presentation 20.0 software with a black Arial font (size 27) on grey background.

### EEG Recording

The 128-channel active EEG actiCHamp setup and BrainVision Recorder, software (BrainProducts, GmbH, Gilching, Germany) were used to investigate the neurophysiological correlates of word learning. The electrodes were applied according to the extended 10–10 system (M1-ext montage by Easycap GmbH, Germany) with FCz as a reference channel, and one EOG electrode was placed under the left eye. 1 kHz sampling rate was used.

The EEG was recorded during a silent reading task. The participants’ attention to the reading task was ensured by use of rare target stimuli (city names; 40 items, repeated twice) randomly dispersed among the main experimental stimuli; novel words (20 items, repeated 10 times); and untrained fillers (60 items, equally composed of real words and orthographically similar pseudowords, repeated 10 times). They were randomly presented (black Arial font (size 24), grey background, 600 ms per word, 1400-ms interstimulus interval with a fixation cross) with the instruction to read all stimuli carefully and press the button (response pad RB-740, Cedrus Corp., San Pedro, CA) with the left index finger each time a city name appeared on the screen.

### EEG Analysis

#### Preprocessing

Custom-built scripts in MATLAB 6.0 (MathWorks Inc., Natick, MA) and the Berlin Brain-Computer Interface (BBCI) toolbox (https://github.com/bbci,GitHub) were used for EEG analysis. First, a band-pass filter between 1 and 45 Hz (2th-order Butterworth filters), down-sampling to 250 Hz sampling rate, and re-referencing to the common average reference, was applied to the raw EEG data. Then, the EEG data were visually inspected to remove artefacts, particularly those associated with muscle activity. Independent component analysis was performed and components associated with blinking and eye movement were removed.

#### Alpha oscillation analysis

We estimated three parameters of alpha oscillations: amplitude, LRTC, and variability (coefficient of quartile variation, CQV).

#### Amplitude

**Figure 2.**
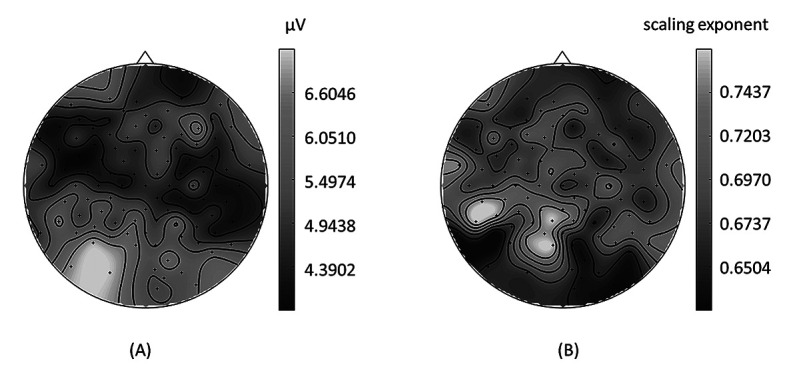
General distribution of amplitude of alpha rhythm (A) and its LRTC scaling exponent (B) averaged for all subjects and conditions

The amplitude (extracted with 8–12 Hz band-pass Butterworth second-order filter) was computed using an analytic signal approach based on the Hilbert transform for each subject and each channel over the entire continuous recording. For each subject, all data from all channels were averaged, so we eventually had one mean value of alpha amplitude for each subject (*Figure 2A*).

#### LRTC

To estimate LRTC, we used detrended fluctuation analysis (DFA) of the amplitude envelope of alpha neuronal oscillations (Kantelhardt, Koscielny-Bunde, Rego, Havlin, & Bunde, 2001; Peng, Havlin, Stanley, & Goldberger, 1995). Note that LRTC refers to the correlation between different time points in EEG activity, not across different spatial locations. Technical details on the use of DFA for the estimation of LRTC in EEG signals can be found in Hardstone et al. (2012). Finally, we had one mean DFA exponent for each subject (*Figure 2B*).

#### CQV

The variability was estimated from the amplitude envelope of alpha oscillations extracted as described above. It was quantified with the coefficient of quartile variation (CQV), a descriptive statistic based on quartiles’ information (Bonett, 2006):


CQV=(Q3−Q1)/(Q3+Q1)

In (1), Q1 and Q3 denote the first (lower) and third (upper) quartiles of the data, respectively. Quartiles are the points that divide any ranked data set into four equal groups. Finally, we had one mean CQV coefficient for each subject.

#### ERP analysis

First, we segmented the preprocessed EEG recording into epochs from –200 ms before the stimulus event (with -200-0 ms interval used as the baseline) to 1000 ms affier that. Second, standard deviation analysis was implemented for each segment using the Berlin BCI toolbox in MATLAB (https://github.com/bbci,GitHub). Since both of the expected ERP components (P200 and N400) could be detected above the fronto-central region, and the frontal and prefrontal cortex play a crucial role in the assimilation of new word forms (Eichenbaum, 2017; Plakke, Romanski, & Kikuchi, 2014), we analyzed ERPs measured above this region to assess their activity in the process of novel word learning. We used averaged ERPs from F1, F2, and Fz electrodes (ROI above frontal/prefrontal cortex) to quantify differences in ERP amplitudes between the groups (see below).

### Learning Outcome Assessment

Five tasks were chosen to assess the success of learning: 1) Free Recall (performed before the EEG task to avoid the impact of the passive reading task in EEG on the novel word recall accuracy); 2) Recognition; 3) Lexical Decision; 4) Semantic Definition; and 5) Semantic Matching (*Table 1*). Microsoft Excel Spreadsheets were used for tasks 1 and 4, and NBS Presentation software for the others (with the same screen and text parameters as in the learning procedure above). The stimulus set and the presentation procedure for the Recognition and Lexical Decision tasks were the same as for the EEG reading task, with the exception that the latter also included target stimuli (city names). The integrative variable General Success was calculated as the mean of z-scores of all task results (accuracy and quality, see *Table 1*).

**Table 1:** Assessing learning outcomes

No	Name	Presented stimuli	Task	Scoring
1	Free Recall	–	To recall all novel word forms without cues	Number of correctly recalled letters (letter strings with fewer than 3 correct letters per word were discarded)
2	Recognition	Novel words, fillers, and controls (words and dowords)	To press ‘yes’ or ‘no’ re- pseu- sponse key, depending on whether the stimulus was presented on the learning stage or not	Number of ‘yes’ answers to novel words
3	Lexical Decision	Novel words, fillers, and controls (words and dowords)	To press ‘yes’ or ‘no’ re- pseu- sponse key, depending on whether the stimulus was a meaningful word or not	Number of ‘yes’ answers to novel words
4	Semantic Definition	Word forms of novel nouns	To type the definition for each novel word	Quality of definition, assessed by four experts, and accuracy of definition — amount of definitions correctly corresponding to the word forms
5	Semantic Matching	Word forms of novel nouns and four options: one appropriate definition, two definitions corresponding to other words, and ‘none of the above’	To choose the correct definition novel	Number of correct choices
6	Integrative variable General Success (calculated as the mean of z-scores of all tasks).				

### Psychological Assessment

To evaluate conceptual thinking abilities, we used the second (Excluding the Word) and third (Analogies) subtests of the Russian version of Amthauer’s IST test (Golovei & Rybalko, 2006). Tolerance of ambiguity was assessed using the MSTAT-I questionnaire (McLain, 1993). Osin (2010) had previously adapted this questionnaire for Russian students and determined the questionnaire’s complex two-dimensional structure. For this study, the dimension of attitude toward ambiguous situations was chosen. Thus, we measured two variables - Preference for ambiguous situations (direct scales), and Acceptance/avoidance of ambiguous situations (inverted scales).

BIS/BAS scales were developed by Carver and White (1994) to measure two motivational systems: a behavioral inhibition system (BIS) corresponding to avoiding aversive outcomes, and a behavioral activation system (BAS) which, in turn, consists of three subscales: Fun-Seeking, Drive, and Reward Responsiveness. Fun-Seeking is associated with impulsivity, whereas other subscales are related to reward sensitivity and reaching goals. In this study, we used the Russian version of the BIS/BAS questionnaire adapted by Knyazev and colleagues (2004).

The psycho-diagnostic techniques were selected based on the existing literature. The two scales of Amthauer’s test measure semantic conceptual abilities, which play a crucial role in academic success (Kholodnaya, Trifonova, Volkova, & Sipovskaya, 2019). BIS/BAS scales, in turn, measure two motivational systems that underlie human behavior and affect (Carver & White, 1994); this is important due to the role of motivation in language learning, as highlighted in the Introduction. MSTAT-1 was selected because of its present use of contextual learning, which requires readiness to act in ambiguous, unclear situations, such as an encounter with previously unknown words within short story-like sets of sentences. The latter resembles a situation when a child is faced with a new word, which is unfamiliar to them at both the word-form and meaning levels.

### Statistical Analysis

Statistical analysis was conducted using IBM SPSS Statistics 26.0 software. The reliability of BIS/BAS and MSTAT-1 was evaluated using Cronbach’s alpha coefficient. High reliability was revealed for Preference of Ambiguous Situations (0.750), Acceptance/Avoidance of Ambiguous Situations (0.869), Drive (0.775), Reward Responsiveness (0.764), and BIS (0.767). However, Cronbach’s alpha was low (0.241) for Fun-Seeking; therefore this subscale was excluded from further analysis.

To minimize the number of variables, psychological and alpha oscillation (amplitude, CQV, and LRTC) parameters were factored using the principal components method with varimax rotation (Kaiser, 1958). Then, correlations between the obtained components and the behavioral task results were calculated using the nonparametric Spearman Rho test.

For paired comparison between groups of subjects with different success levels, the sample was divided into two main groups: less successful learners (LSL), who scored General Success values less than M– 0.25σ (17 people, 58.82% male), and more successful learners (MSL), who had General Success scores above M + 0.25σ (10 people, 30% male); three participants with intermediate values were excluded from further analysis. The LSL and MSL groups were compared with Pearson χ2 and Fisher’s exact tests for the nominative variable (Gender) and U Mann-Whitney for the others.

Between-group (MSL vs. LSL) comparison of ERP amplitudes, computed in 8-ms bins between 0 and 800 ms, was done using the Wilcoxon test for independent samples (two-tailed) implemented in the MATLAB environment. FDR correction for multiple comparison was implemented.

## Results

### Socio-demographic Characteristics

Comparison between the LSL and MSL groups showed that they did not differ statistically in Gender (χ2 = 2.095, p (Fisher’s exact test) = 0.236), Age, and Handedness, but more successful word learners had more years of Education (*Table 2*).

**Table 2:** Socio-demographic characteristics (Mean ± SE or % of total N)

Variable	Sample N = 30	MSL N = 17	LSL N = 10	LSL/MSL (U Mann-comparison Whitney)	Correlation with (General Spearman’s Success Rho)
Age	23.43± 0.74	23.10±0.62	23.76±2.73	Z = –0.45, p = 0.675	r = 0.006, p = 0.976
Handedness	70.83±4.52	71.91±8.60	69.64±18.06	Z = –0.23, p = 0.824	r = –0.105, p = 0.579
Education	14.88± 0.40	16.35±0.58	14.53±0.46	Z = –2.30, p = 0.023*	r = 0.306, p = 0.100

Note. LSL = less successful learners. MSL = more successful learners. * p≤0.05

### Factor Analysis

The Kaiser-Meyer-Olkin coefficient was 0.510, which indicated that the factor analysis was appropriate for these data. The Bartlett’s test of sphericity showed that the variables had correlations with each other (p = 0.004, chi-square = 74.179, df = 45); thus, they were suitable for structure detection. The principal components method with varimax rotation extracted five factors with a cumulative contribution rate of 80.436% (*Table 3*).

**Table 3:** Factor analysis — Rotated component matrix

Variables	Factors				
	1	2	3	4	5
	Physiological Indicators of Low Attention Concentration	Tolerance of Ambiguity	Persistence in Concep- tual Thinking	Reward/ Punishment Sensitivity	Cognitive Processing Neuro-dynamics
Amplitude of alpha oscillation	0.909				
CQV	0.847				
Preference for ambigu- ous situations		0.895			
Acceptance/avoidance of ambiguous situations		0.804			
BAS — Drive			0.851		
Analogy (Amthauer)			0.759		
BAS — Reward Responsiveness				0.874	
BIS				0.770	
Excluding the word (Amthauer)					0.836
LRTC					–0.690

*Note. Coefficients less than 0.4 are not shown*

### Correlation between Factors and Success

The interrelationships between word learning success (both as an integrative variable and for each task separately) and the five factors were analyzed using a non-parametric Spearman’s rank correlation test (*Table 4*: p-values reported without correction for multiple comparisons; the correlations which survived FDR corrections for multiple comparisons are **underlined**).

**Table 4:** Correlations between factors and learning outcomes

	Free Recall	Recognition	Lexical Decision	Semantic Definition (Accuracy)	Semantic Definition (Quality)	Semantic Matching	General Success
Physiological Indicators of Low Attention Concentration	r = –0.188,	r = –0.504**,	r = –0.177,	r = 0.230,	r = 0.136,	r = 0.136,	r = –0.127,
	p = 0.319	p = 0.004	p = 0.351	p = 0.221	p = 0.472	p = 0.851	p = 0.502
Tolerance of Ambiguity	r = –0.205,	r = –0.093,	r = –0.105,	r = –0.401*,	r = –0.113,	r = –0.160,	r = –0.383*,
	p = 0.276	p = 0.626	p = 0.580	p = 0.028	p = 0.551	p = 0.399	p = 0.037
Persistence in Conceptual Thinking	r = 0.496**,	r = 0.006,	r = 0.072,	r = 0.558**,	r = 0.303,	r = 0.470**,	r = 0.543**,
	p = 0.005	p = 0.974	p = 0.706	**p = 0.001**	p = 0.103	p = 0.009	**p = 0.002**
Reward / Punishment Sensitivity	r = 0.107,	r = 0.183,	r = –0.210,	r = –0.113,	r = –0.075,	r = –0.081,	r = –0.149,
	p = 0.574	p = 0.334	p = 0.265	p = 0.553	p = 0.695	p = 0.670	p = 0.431
Cognitive Processing Neurodynamics	r = 0.053,	r = 0.488**,	r = –0.15,	r = 0.186,	r = 0.095,	r = 0.144,	r = 0.272,
	p = 0.78	p = 0.006	p = 0.428	p = 0.326	p = 0.619	p = 0.446	p = 0.146

Note. * p < .05. ** p < .01

The Reward/Punishment Sensitivity factor did not interact significantly with any of the task results. The factors Physiological Indicators of Low Attention Concentration and Cognitive Processing Neurodynamics significantly correlated with Recognition scores. Tolerance of Ambiguity negatively correlated with General Success and the accuracy of Semantic Definition. The Persistence in Conceptual Thinking factor had positive interrelationships with accuracy scores on three tasks (Free Recall, Semantic Definition, and Semantic Matching) and the composite General Success measure.

Thus, we found a relationship between psychological and neurophysiological characteristics at the basic level of word acquisition related to surface word-form memory (as measured by the recognition task). Moreover, significant correlations were demonstrated for factors with high factor loadings of alpha oscillation parameters. The connection between psychological variables and word-learning success, in turn, concerned the acquisition of novel semantics. The Persistence in Conceptual Thinking factor was revealed as the most influential variable in word-learning success. Moreover, correlations between this factor and behavioral task results (Semantic Definition accuracy and General Success) stayed fully or marginally significant even after FDR corrections (adjusted p = 0.035 and 0.068, respectively). This factor depicts persistence as a personal trait and sign of conceptual thinking capacity.

### Between-group Comparison of ERPs to Novel Words

**Figure 3.**
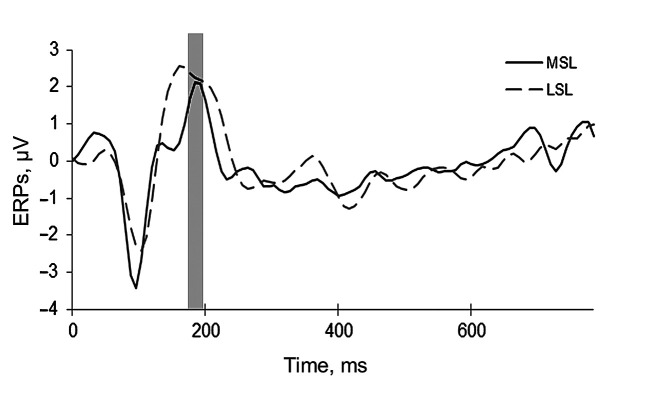
Average frontal ERPs on novel words for groups of more (MSL) and less (LSL) successful learners. P2 time window, where the differences were most expressed, is highlighted

To assess overall differences in brain activity between the LSL and MSL groups, amplitudes of ERPs over the frontal sensor ROI were compared across 800 ms after stimulus onset, using the two-tailed independent-sample Wilcoxon test step in bins of 8 ms. Significant differences were found at 153–161 ms (Z = –1.98, p = 0.047 uncorrected), 161–169 ms (Z = –2.54, p = 0.011 uncorrected), and 169-177 ms (Z = –2.08, p = 0.037 uncorrected) from the stimulus onset: more successful learners had lower frontal ERP amplitude (*Figure 3*). None of these results, however, survived after FDR corrections.

## Discussion

Our study aimed to investigate success in novel word acquisition in connection with learners’ attention level (measured as amplitude of P200 component and alpha oscillation parameters), verbal cognitive abilities, motivation, and tolerance of ambiguity. The results showed that both psychological and physiological variables interacted with word learning success. Socio-demographic characteristics, on the other hand, did not correlate with this parameter. However, groups of more and less successful learners differed significantly in the number of years of education; more successful participants had more studying experience. This may suggest that the experience of learning novel material during formal education (and possibly of being tested on it) could positively influence the ability to learn novel words. This may be due to an increase in the number of vocabulary learning strategies that accelerate vocabulary growth (Nie, 2017), and more general cognitive learning strategies which help in acquiring information efficiently (Kostromina & Dvornikova, 2016).

The two groups differed in frontal ERP only in the time window around 150-180 ms. This wave likely corresponds to the P200 component, whose amplitude is known to negatively correlate with the level of attention (Crowley & Colrain, 2004) and to reflect inhibition of irrelevant information (Meghdadi et al., 2021; Zhao, Zhou, & Fu, 2013). Thus, more successful word learners were more attentive than others and were better at suppressing irrelevant inputs. Interestingly, this result was found during the reading task *after* the training session; we may hypothesize that the same trait was also expressed during the learning per se.

As for P200 as a marker of orthographic encoding, in a recent study it was shown that novel (firstly seen) words elicited lower P200 than previously known ones; however, this effect had disappeared after a short phonological training session (Bermúdez-Margaretto et al., 2020). The fact that the EEGs were recorded after the training session that provided an equal number of encounters with novel words for both groups of participants, suggests that the presence differences in P200 amplitude more likely reflected the level of attention than the depth of orthographic processing during visual recognition.

We also expected to find differences in the N400 amplitude, since this component is known to reflect lexico-semantic properties of verbal (and other meaningful) stimuli and their integration into a person’s lexicon. However, no N400 effects were found, and no marked deflection was recorded in the N400 range, as can be seen in *Figure 3*. The absence of significant differences in the amplitude of the N400 component probably stems from the absence of context in the passive word reading task applied in the EEGs (Abel, Schneider, & Maguire, 2018; Bermúdez-Margaretto, Beltrán, Cuetos, & Domínguez, 2018).

Factors with high factor loadings of alpha oscillations parameters had significant correlations with accuracy in the Recognition task only. Thus, better recognition of novel words appeared to relate to higher attention concentration as reflected in the amplitude of alpha oscillations (Bastiaansen et al., 2002; Klimesch, 1999), and to excitation/inhibition balance as reflected in LRTC (Beliaeva et al., 2019). This might indicate that the perception of novel words required high involvement of the executive control systems, upregulating the level of attention.

We also found that the General Success and Accuracy of the Semantic Definition task negatively correlated with the ambiguity tolerance measure. The least ambiguity-tolerant students had better performance in the acquisition of word meanings. These results contradict previous studies that indicated a positive influence of ambiguity tolerance on L2 acquisition. However, there is evidence that the correlation of ambiguity tolerance with academic success could vary (Osin, 2010). Dealing with a large number (here, 20) of unknown words could elicit anxiety in learners with low levels of ambiguity tolerance. That, in turn, could have motivated them to resolve the ambiguities in order to understand the meanings of new words better, fostering better learning. Interestingly, the Quality variable (Semantic Definition task) showed no correlations with psychophysiological factors. It seems that the wording of definitions is a complex cognitive process that connects with other psychological and neurophysiological parameters that were not included in the study; alternatively, the measure applied here to estimate definitions’ quality may not have been sufficiently precise or sensitive to demonstrate such connections.

The Persistence in Conceptual Thinking factor positively correlated with the results of three tasks and the integrative variable General Success. This result implies that logical conceptual thinking ability and persistence in reaching goals support the acquisition of both the forms and meanings of novel words. Moreover, only this factor stayed significantly correlated with learning outcomes after FDR corrections. Thus, our study showed that psychological features had stronger interrelationships with word acquisition success than neurophysiological ones. It appears necessary to continue interdisciplinary investigation of the word acquisition process to better elucidate connections between its psychological, behavioral, and neurophysiological aspects, which remain poorly understood (Mkrtychian et al., 2019).

## Conclusion

This study investigated psychological and neurophysiological factors involved in successful word acquisition. The results have shown a range of psychological features related to performance in semantic tasks on novel word comprehension, whereas neurophysiological variables seem to be linked to successful recognition of newly acquired word forms. The more successful group of learners also showed lower P200 amplitude than their less successful peers, suggesting differences in the level of attention, which may have contributed to better learning.

## Limitations

Whereas the present study has produced novel results on psychological and neuro-physiological factors related to successful word acquisition, it still has several confounds and limitations that necessitate caution in interpreting its results. The relatively small sample size restricted the number of variables that could be analyzed. Thus, only 10 parameters were used in the research. Crucially, only some of the alpha-band parameters were explored, while neither brain oscillations in other bands — such as beta and theta, which are closely related to memory and learning (Herweg, Solomon, & Kahana, 2020), including verbally (Bakker, Takashima, van Hell, Janzen, & Mc-queen, 2015) — nor ERPs above other brain areas, were examined.

Moreover, whereas relationships between word learning success and psycho-physiological factors could vary depending on age (Kamal, Morrison, Campbell, & Taler, 2021; Morrison & Taler, 2020), the results of the study are restricted to the young sample used in the experiments (18-35 years old). Finally, all participants were monolingual Russian speakers, and the novel words were orthographically and phonologically native-like, and were presented in L1 sentence context. Thus, the results of the study may not be generalizable to other languages or L2 learning without further investigation.
